# Initial Experience of Intra-Arterial Chemotherapy Using a Novel External Carotid Arterial Sheath System Combined with Radiotherapy and Systemic Chemotherapy for Locally Advanced Tongue Cancer

**DOI:** 10.3390/cancers14225529

**Published:** 2022-11-10

**Authors:** Miwako Nomura, Nobukazu Fuwa, Shintaro Ito, Yutaka Toyomasu, Akinori Takada, Daisuke Kobayashi, Tomohito Fuke, Masanori Taniguchi, Noriko Ii, Junji Uraki, Hiroyuki Yamada

**Affiliations:** 1 Department of Radiation Oncology, Ise Red Cross Hospital, 1-471-2 Funae, Ise 516-8512, Japan; 2 Department of Radiation Oncology, Central Japan International Medical Center, 1-1 Kenkounomachi, Minokamo 505-8510, Japan; 3 Department of Medical Technology, Ise Red Cross Hospital, 1-471-2 Funae, Ise 516-8512, Japan; 4 Department of Radiology, Mie University Hospital, 2-174 Edobashi, Tsu 514-8507, Japan; 5 Department of Head and Neck Otorhinolaryngology, Ise Red Cross Hospital, 1-471-2 Funae, Ise 516-8512, Japan; 6 Department of Medical Oncology, Ise Red Cross Hospital, 1-471-2 Funae, Ise 516-8512, Japan; 7 Department of Radiology, Ise Red Cross Hospital, 1-471-2 Funae, Ise 516-8512, Japan

**Keywords:** intra-arterial chemotherapy, tongue cancer, radiation therapy

## Abstract

**Simple Summary:**

Surgery is the current standard for locally advanced tongue cancer. However, substantial impairment may occur even after treatment. Therefore, more effective and less toxic treatment strategies are needed to avoid a reduction in the patient’s quality of life. This study aims to evaluate the efficacy and safety of a novel device that we have developed for retrograde intra-arterial chemotherapy (IACT) in patients with locally advanced tongue cancer. Our study demonstrates that the 3-year overall survival and progression-free survival were 81.6% and 74.2%, respectively. These results are not inferior to those of surgery, and no complications (e.g., cerebral infarction or catheter-related infection) have been associated with IACT were observed.

**Abstract:**

We retrospectively evaluated the safety and effectiveness of an external carotid arterial sheath (ECAS) for intra-arterial chemotherapy (IACT) for locally advanced tongue cancer. Thirty-one patients with the Union for International Cancer Control’s 8th TNM stage III–IV tongue cancer underwent IACT using the ECAS combined with RT and systemic chemotherapy with either cisplatin and fluorouracil (FP) or docetaxel, cisplatin, and fluorouracil (TPF) between October 2015 and February 2021. The ECAS was inserted retrogradely via the superficial temporal artery, and the tip was placed in the external carotid artery between the maxillary and facial arteries. A microcatheter was inserted into each tumor-feeding artery through the ECAS under fluoroscopy, wherein cisplatin 50 mg/m^2^ was administered. IACT was performed weekly with neutralization using sodium thiosulfate. Complete response of the primary lesion was achieved in 28/31 (90%) patients. The median follow-up for all patients was 39 months. The 3-year overall survival, progression-free survival, and local control rates were 81.6%, 74.2%, and 83.4%, respectively. Grade 3 and greater toxicities included oral mucositis (45%), neutropenia (39%), nausea (13%), anemia (10%), thrombocytopenia (10%), dry mouth (10%), and fever (3%). There were no severe complications associated with IACT. In conclusion, the ECAS is feasible and effective for locally advanced tongue cancer.

## 1. Introduction

Tongue cancer accounts for approximately 1% of all malignant neoplasms worldwide [1]. Although the common risk factors are smoking and alcohol abuse, the incidence is progressively increasing among young adults without chronic exposure to such risk factors [2,3]. Surgery followed by adjuvant radiotherapy (RT) with or without chemotherapy is the current standard for locally advanced tongue cancer; however, substantial impairment even after treatment still occurs [4,5]. Therefore, more effective and less toxic nonsurgical strategies are urgently needed to avoid a reduction in the patient’s quality of life.

IACT for head and neck cancer was made possible through advancements in angiographic techniques in the 1990s. In IACT, two major approaches to catheterization are used [6,7]. In one approach, a catheter is anterogradely inserted through the femoral artery using the Seldinger technique, whereas in the other, the catheter is retrogradely inserted from the superficial temporal artery (STA) [4,8,9]. Although the anterograde approach enables a catheter to be inserted into several tumor-feeding arteries, the incidence of procedure-related neurological complications, which was reported to be approximately 1–3%, has been a major problem [10,11]. In contrast, the conventional retrograde approach via the STA is rarely associated with neurological complications; however, its limitation is that only one artery can be used [6].

As the tongue is mainly fed by the lingual (LA) and facial arteries (FA), Mitsudo et al. developed a two-channel method in which one catheter is inserted into the LA via the occipital artery (OA) and another is inserted into the FA via the STA [12,13]. Although this method allows drugs to be simultaneously administered into the LA and FA, the procedure is complicated, and only specific arteries can be used.

The external carotid arterial sheath (ECAS), which is a novel device made from a 5-Fr heparinized catheter (ANTHRON P-U Catheter, Toray Medical Co., Ltd., Tokyo, Japan), functions similarly to a typical sheath placed on the femoral artery. The ECAS enables angiographers to simultaneously select several tumor-feeding arteries with a microcatheter when performing retrograde IACT [14,15]. This is the first clinical study that assesses the safety and effectiveness of IACT using the ECAS system combined with chemoradiotherapy (CRT) for locally advanced tongue cancer.

## 2. Materials and Methods

### 2.1. Eligibility Criteria

Patients with primary tongue cancer who met the following criteria were enrolled in this retrospective study: presence of pathologically confirmed T3–4 N0–3 M0 squamous cell carcinoma of the tongue; refusal to undergo surgery or inoperable; Eastern Cooperative Oncology Group (ECOG) performance status (PS) 0–2; aged 20–76 years; sufficient bone marrow function (white blood cell count > 3000/mm^2^ and platelet count > 100,000/mm^2^); absence of liver, kidney, heart, and lung abnormalities; and untreated tongue cancer. The presence of curable, active secondary cancer at the beginning of treatment was allowed.

The extent of the primary tongue lesion was evaluated by magnetic resonance imaging (MRI), computed tomography/positron emission tomography (CT/PET) with 2-[fluorine-18]-fluoro-2-deoxy-D-glucose (FDG-PET/CT), visual examination, and palpation. The absence of distant metastasis was investigated by CT/PET.

The local institutional research board approved this study (27-6), and informed consent was obtained from each participant.

### 2.2. Treatment Procedure

**Figure 1:**
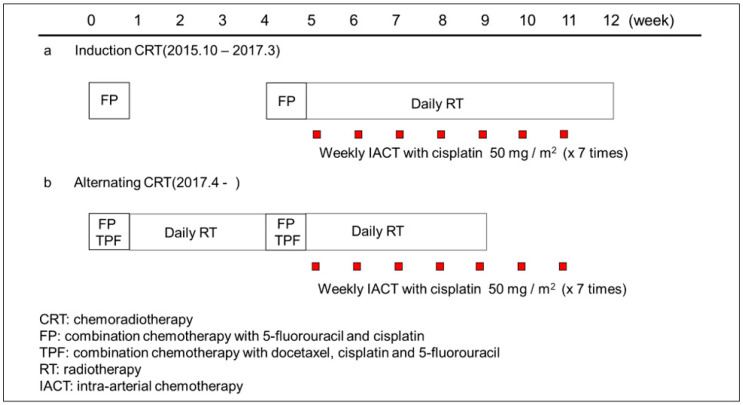
Treatment scheme. (**a**) Until March 2017, patients received two courses of induction chemotherapy (cisplatin and fluorouracil [FP]), followed by daily radiotherapy (RT) combined with weekly intra-arterial chemotherapy (IACT). (**b**) From April 2017, patients first received alternating chemoradiotherapy, which comprised two courses of chemotherapy (regimen FP or docetaxel, cisplatin, and fluorouracil [TPF]) and daily RT followed by concurrent RT with weekly IACT.

The treatment scheme is shown in Figure 1. From October 2015 to March 2017, the basic treatment was two courses of induction chemotherapy followed by weekly retrograde IACT with cisplatin combined with seven weeks of RT. However, since two patients had progression of disease during the drug holiday following induction chemotherapy, we revised the induction chemotherapy to alternating chemotherapy to ensure continuous antitumor treatment beginning April 2017. Specifically, patients received an initial course of chemotherapy followed by large-field radiotherapy with intensity-modulated radiation therapy (IMRT), including the prophylactic area. Patients then received a second course of chemotherapy, followed by weekly IACT with cisplatin combined with sequentially shrinking field radiotherapy with IMRT.

### 2.3. Chemotherapy

From October 2015 to March 2017, patients received induction chemotherapy (regimen FP) consisting of cisplatin 80 mg/m^2^/2 h on day 1 and 5-fluorouracil 800 mg/m^2^/24 h for 5 days (days 1–5). From April 2017, patients aged ≤ 60 years received chemotherapy (regimen TPF) comprised of docetaxel 60 mg/m^2^/1 h and cisplatin 60 mg/m^2^/2 h on day 1, and 5-fluorouracil 600 mg/m^2^/24 h for 5 days (days 1–5).

### 2.4. Radiotherapy

Radiotherapy was planned after appropriate immobilization using a thermoplastic mask and CT-based techniques. IMRT was performed five times a week by irradiating 1.8 Gy of photon beam in a fraction using a 6-MV linear accelerator. The gross tumor volume (GTV) was determined by visual examination, palpation, and imaging modalities, including CT, MRI, and/or PET-CT. The clinical target volume (CTV) was defined as the GTV plus a margin of 3–5 mm to cover microscopic disease, and the planning target volume was defined as the CTV plus a margin of 3 mm. In the absence of cervical lymph node metastasis, the CTV contained the primary site and neck levels I–III on the ipsilateral side. The dose was delivered in 36 Gy/20 fractions, and boost irradiation to the primary tumor was delivered in 18 Gy/10 fractions. When cervical lymph node metastasis was present, the CTV consisted of the primary site and neck levels I–IV on the ipsilateral side (N1) or neck levels I–V on the bilateral side (N2–3). The dose was delivered in 36 Gy/20 fractions, and boost irradiation to the primary tumor and metastatic lymph nodes was delivered in 18 Gy/10 and 32.4 Gy/18 fractions, respectively. In principle, we restricted the radiation dose at the primary site to 54 Gy in order to avoid radiation-induced late adverse events, especially osteoradionecrosis of the mandible.

### 2.5. Intra-Arterial Chemotherapy

As previously reported [14], the anterior ear on the affected side was incised under local anesthesia to expose the STA. Under fluoroscopy, the ECAS (10 cm long and 5-Fr [outer diameter], Toray Medical Co., Ltd., Tokyo, Japan) was inserted retrogradely through the STA, and its tip was placed between the maxillary artery and the FA. The ECAS remained indwelling during the 6–10-week course of IACT. When the lesion involved the contralateral side, another ECAS or 5-Fr heparinized catheter (ANTHRON PU catheter, Toray Medical Co., Ltd., Tokyo, Japan) was inserted into the contralateral side for bilateral IACT.

**Figure 2:**
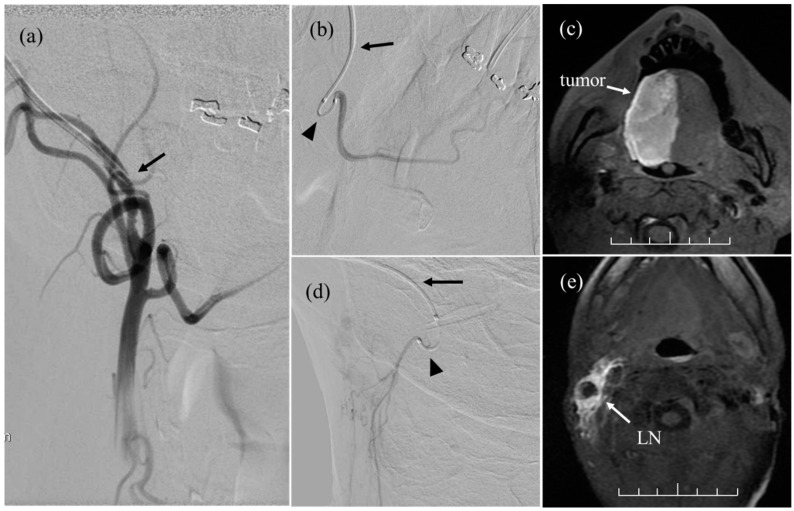
(**a**) External carotid arteriography obtained by administering contrast media via the external carotid arterial sheath (ECAS) on digital subtraction angiography (DSA). Arrow: tip of ECAS. (**b**) Superselective lingual arteriography via a steerable microcatheter through the ECAS on the DSA. Arrow: tip of ECAS; arrowhead: a steerable microcatheter. (**c**) Magnetic resonance image showing the injected contrast agent via the right lingual artery. (**d**) Arteriography of a branch from the ECA to the metastatic lymph nodes on the DSA. (**e**) Magnetic resonance image showing the injected contrast agent via the direct branch from the ECA. Scale bar.

Each cycle of weekly IACT was performed under fluoroscopic guidance. Before the procedure, the ECAS was thoroughly disinfected with povidone-iodine. First, contrast media was injected through the ECAS, and a roadmap was created to identify the position of the target arteries using digital subtraction angiography (DSA; Figure 2a). Referring to the roadmap, the tumor-feeding arteries were selected by a steerable microcatheter (SwiftNinja^®^ Steerable Microcatheter, Merit Medical System Inc., Parkway South Jordan, UT, USA) inserted through the ECAS [15]. Figure 2b shows the superselective lingual arteriography via the microcatheter on DSA. Cisplatin 50 mg/m^2^ was manually injected into each tumor-feeding artery at a rate of 2.5 mg/min. The injection time per artery was 10–15 min.

The distribution of cisplatin delivered to each artery was determined based on the findings of the MRI and the extent of the blue dye. Figure 2c shows the MR image of the injected contrast medium via the right lingual artery. Sodium thiosulfate was used as a cisplatin-neutralizing agent and was intravenously administered 1 h prior to cisplatin administration at a dose of 0.4 g/1 mg of cisplatin over 8 h. Although the total number of IACT sessions was standardized to seven, this was revised according to the therapeutic response.

### 2.6. Patient Assessments

For this analysis, all patients were staged according to the Union for International Cancer Control (UICC) TNM Classification, 8th edition. Acute toxicities were assessed according to the Common Terminology Criteria for Adverse Events Version 4.0. The antitumor effects (primary effects) of the treatment were evaluated based on the results of an MRI and CT/PET performed 2–3 months after the completion of treatment and according to the RECIST criteria. Disease progression after treatment was evaluated every 1–2 months for the first year and every 3–4 months thereafter.

Overall survival (OS) was measured from the first day of treatment to the date of death from any cause. Progression-free survival (PFS) was calculated up to the first confirmed day of locoregional progression or recurrence, the first detection of distant metastasis, or death from any cause. Local control (LC) was calculated up to the day of confirmation of primary tumor growth.

### 2.7. Statistical Analysis

The primary study endpoint was the 3-year OS, and the secondary endpoints were the 3-year PFS and LC, the safety and effectiveness of the ECAS system, including adverse effects related to the procedure, and arterial selectivity by the microcatheter. The OS, PFS, and LC curves were estimated using the Kaplan–Meier method, and risk factors associated with OS, PFS, and LC were analyzed by the Cox proportional hazards model. Differences were considered significant when the *p*-value was <0.05. Data analysis was performed using the EZR software (version 3.6.1, Saitama Medical Center, Jichi Medical University, Saitama, Japan) [16].

## 3. Results

### 3.1. Patient Characteristics

Between October 2015 and February 2021, 33 patients with locally advanced tongue cancer met the eligibility criteria. Of these, two were excluded due to performing IACT via a femoral access. They received IACT via femoral access only once or twice because the tumor almost disappeared with systemic CRT before IACT. The remaining 31 patients (female, *n* = 8; male, *n* = 23; age range = 25–76 years; median age, 49) who underwent IACT using the ECAS system combined with CRT were included in the final analysis. Table 1 shows the clinical characteristics of the subjects. All patients had an ECOG PS of 0–2 and histologically proven squamous cell carcinoma. Of the 31 patients, 5 (16%) had stage III, 24 (77%) had stage IVA, and 2 (7%) had stage IVB disease. Meanwhile, two (6%) patients had inoperable disease, and four patients had synchronous double cancer (one each in the esophagus, stomach, colon, and breast).

The ECAS was positioned in the ECA bilaterally and ipsilaterally in 23 patients and 8 patients, respectively. Of these eight patients, a 5-Fr heparinized catheter (ANTHRON P-U Catheter, Toray Medical Co., Ltd., Tokyo, Japan) was positioned in the contralateral LAs in two patients and in the contralateral ECA in one patient. A cumulative total of 234 cycles of IACT were performed under fluoroscopy (3–14 cycles per each patient; median, 7 cycles), and the microcatheter was inserted into 112 arteries in the 31 patients (2–9 arteries per patient; median, 4). Arteries selected by the microcatheter were as follows: 27 ipsilateral LAs, 27 ipsilateral FAs, 5 ipsilateral OAs, 4 ipsilateral common trunks, 3 ipsilateral small branches from the ECA to the upper jugular node metastases (Figure 2d,e), 2 ipsilateral ascending pharyngeal arteries, 1 ipsilateral superior thyroid artery, 20 contralateral LAs, 17 contralateral FAs, 2 contralateral common trunks, 2 contralateral OAs, 1 contralateral small branch from the ECA to upper jugular node metastases, and 1 contralateral superior thyroid artery. The cumulative total number of arteries selected by the microcatheter in 234 cycles of IACT was 858. The OA was selected to treat upper jugular node metastases. In patients with tumors invading the retromolar trigone, drugs were administered to the maxillary arteries via the ECAS. The successful arterial selectivity rate of the microcatheter was 95% (818/858). Unselectable arteries included the LAs branching off from the common trunk in six patients. The median dose of cisplatin was 565 mg per patient (range: 270–830 mg). One patient with a treatment-resistant tumor was administered additional docetaxel at a total dose of 75 mg in four fractions. The median radiation dose to the primary tumor and metastatic lymph nodes was 56 Gy (range: 36–70 Gy) and 68.4 Gy (range: 48.6–77.6 Gy).

Of the 31 patients, 16 (52%) received systemic FP chemotherapy, whereas the remaining 15 (48%) received the TPF regimen. Additionally, 25 (81%) patients received a second course of chemotherapy. The remaining 6 (19%) patients received only one course of systemic chemotherapy due to the following reasons: rapid tumor growth in 2 (urgent arterial injection was needed), severe oral mucositis in 1, bone marrow toxicity in 1, hyponatremia in 1, and shortening the treatment period of tongue cancer for surgery of gastric cancer in 1.

### 3.2. Response and Survival

**Figure 3:**
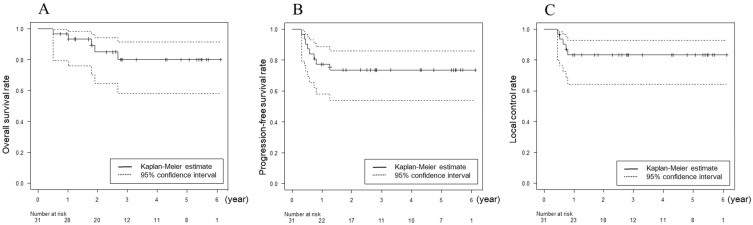
(**A**) Overall survival, (**B**) progression-free survival, and (**C**) local control rates analyzed by the Kaplan–Meier method.

As for the primary effect on the tongue, complete (CR) and partial (PR) responses were achieved in 28 (90%) and 3 (10%) patients, respectively. As for the primary effect of the cervical lymph node metastases, of the 26 patients with cervical lymph node metastases, 19 (73%) achieved clinical CR, whereas 7 patients underwent neck dissection. The median follow-up duration was 39 months (range: 15–79 months) for all patients as of the end of May 2022. Relapse was detected in eight patients during the follow-up: at both the primary site and cervical lymph nodes in four, the primary site alone in one, cervical lymph nodes alone in one, and distant metastases in two. Of the five patients with relapse at the primary site, four had recurrence within the extent of drug perfusion by IACT, and one had recurrence at the lateral oropharyngeal wall, which was outside of the drug perfusion area. Salvage therapies for the six patients with locoregional recurrence were as follows: re-irradiation with IACT followed by nivolumab in three; subtotal glossectomy and bilateral neck dissection in one; partial glossectomy in one, and nivolumab alone in one. During the follow-up period, 5 patients died: 3 died of local failure, 1 died of cervical neck failure, and 1 died of lung metastasis; 25 patients have been alive without disease, whereas 1 was lost to follow-up. The 3-year OS, PFS, and LC were 81.6% (95% CI, 60.0–92.0%), 74.2% (95% CI, 55.0–86.2%), and 83.4% (95% CI, 64.7–92.7%), respectively (Figure 3).

### 3.3. Toxicity

Toxicities are shown in Table 2. The major acute adverse events grade 3 and higher were as follows: oral mucositis (14 patients, 45%), neutropenia (12 patients, 39%), nausea (4 patients, 13%), anemia (3 patients, 10%), thrombocytopenia (3 patients, 10%), dry mouth (3 patients, 10%), and fever (1 patient, 3%). There were no complications associated with IACT (e.g., cerebral infarction, catheter-related infection). No late adverse events were observed during the follow-up period.

### 3.4. Factors Related to OS, PFS, and LC

On univariate analysis, the number of systemic chemotherapy courses (*p* = 0.019) was significantly associated with PFS (Table 3). On multivariate analysis, no factor was significantly associated with PFS (Table 4).

### 3.5. Relationship between the Tumor Volume and Cumulative Cisplatin Dose of CDDP from IACT

**Figure 4:**
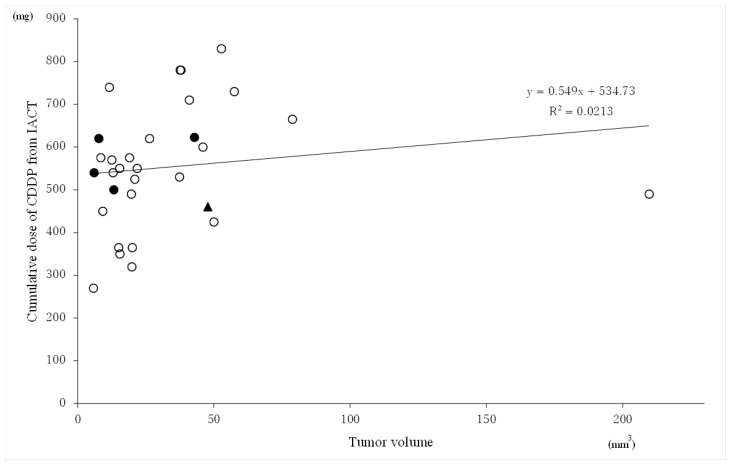
Scatter plot showing the tumor volume and total cumulative dose of CDDP administered with intra-arterial chemotherapy (IACT). Open circles are local control cases, closed circles are local recurrent cases within the perfusion area of IACT, and the closed triangle is a case of local recurrence outside the perfusion area of IACT. The tumor volume and total dose of CDDP were not correlated.

Figure 4 shows the scatter plot between the tumor volume and cumulative cisplatin dose from IACT related to tumor control. Neither the tumor volume nor the cumulative cisplatin dose from IACT were statistically significantly correlated with tumor control.

## 4. Discussion

Several studies have demonstrated that outcomes of oral cavity SCC treated with definitive CRT remain unsatisfactory [17,18]. In a randomized trial comparing surgery and adjuvant RT versus concurrent CRT in patients with advanced head and neck cancer, the subset analyses demonstrated that survival was significantly poorer in patients with oral cavity SCC who underwent CRT than in those who underwent surgery and RT (12% versus 68%, 5-year disease-specific survival; *p* = 0.038) [18]. Another comparative analysis also showed that the 5-year OS after definitive CRT in resectable stage III–IV tongue SCC was significantly inferior to that after primary surgery with adjuvant RT or CRT (20.4% vs. 57.0%) [19]. Therefore, surgery has been the standard treatment for locally advanced tongue cancer.

Patients with stage III–IV tongue SCC demonstrated excellent outcomes following IACT (Table 5). Mitsudo et al. reported that the 3-year OS and locoregional control rates were 81.5% (III, 94.7%; IV, 64.9%) and 80.3% (III, 89.7%; IV, 72.1%), respectively, for 95 patients who underwent daily retrograde IACT with the two-channel method combined with RT [20]. Meanwhile, Takayama et al. reported that the 3-year OS, PFS, and LC rates were 87.0%, 74.1%, and 86.6%, respectively, for 33 patients who underwent weekly conventional retrograde IACT combined with proton beam therapy and systemic chemotherapy [21]. The present study demonstrated that the 3-year OS, PFS, and LC were 81.6%, 74.2%, and 83.4%, respectively, for 31 patients who underwent weekly IACT using the ECAS system combined with CRT. Our treatment results were in correspondence with these results, indicating that IACT can be used to treat advanced tongue cancer.

In a randomized phase 3 trial comparing intra-arterial versus intravenous CRT for advanced head and neck cancer, no differences in locoregional control and OS were observed between the two arms [29]. However, two concerns regarding the trial have been pointed out: (1) as the technique used to deliver intra-arterial infusions lacked sophistication, an insufficient drug dose might have been administered to the targeted tumor volume; and (2) as the majority of the primary sites involved the oropharynx (64%), there may have been cases caused by the human papilloma virus, for which treatment by conventional CRT is preferable. Therefore, the selection of the subjects was considered to be a major problem [30].

From an interventional standpoint, Mitsudo et al. performed retrograde IACT using two channels [20]. Although this method allows for daily concurrent CRT, its limitation is that the selectable arteries are only two of several feeding arteries. Additionally, the surgical procedure is complicated, especially catheterization via the OA, because the OA is anatomically located in a deep layer under the sternocleidomastoid muscle. The methods that Takayama et al. performed were either that the catheter was placed into the ECA via the STA or that the catheter was first positioned into the LA and its tip was subsequently positioned into the ECA to cover the entire tumor bed [21]. In our method, retrograde IACT was achieved by placing a microcatheter introduced through the ECAS, which was indwelled into the ECA via the STA, enabling the delivery of drugs to several tumor-feeding arteries. In the present study, all main branches of the ECA were successfully selected by the microcatheter.

Regarding complications associated with the procedure, Takayama et al. and Mitsudo et al. reported that catheter-related infections occurred in 12% (4/33) and 3% (4/129) of patients, respectively; however, none was observed in the present study despite long-term indwelling of the ECAS (median, 2 months), which may be attributed to the minimal ECAS length inside the artery (5 cm) [20,21]. The incidence of procedure-related neurological complications, which is a serious side effect of IACT via femoral access, was not observed in our method. Given that the procedure is completed within the ECA, the risk of cerebral infarction in our procedure is low.

From a pharmacokinetic standpoint, Takayama et al. performed retrograde IACT by continuously injecting cisplatin at a dose of 20–40 mg/m^2^ over 5 h [21]. In contrast, our method required manual injection of cisplatin 50 mg/m^2^ into each tumor-feeding artery at a rate of 2.5 mg/min under fluoroscopy. The injection time per artery was 10–15 min, which may be a disadvantage because cisplatin causes cytotoxicity in a dose- and time-dependent manner [31,32].

In the present study, IACT was combined with two courses of either neoadjuvant or alternating high dose systemic chemotherapy consisting of cisplatin 80 mg/m^2^/2 h on day 1 and 5-fluorouracil 800 mg/m^2^/24 h for 5 days (days 1–5) or docetaxel 60 mg/m^2^/1 h, cisplatin 60 mg/m^2^/2 h on day 1, and 5-fluorouracil 600 mg/m^2^/24 h for 5 days (days 1–5) because our previous study demonstrated that the use of systemic chemotherapy was a significant prognostic factor [6]. Several meta-analyses have demonstrated that patients with head and neck cancer who underwent induction chemotherapy had a decreased distant metastasis rate of approximately 7% and a better PFS compared with those who underwent concurrent CRT [33,34]. In this and Takayama’s studies, distant metastases occurred in 6% (2/31 and 2/33, respectively) of patients regardless of whether they underwent neoadjuvant or alternating high-dose systemic chemotherapy in addition to IACT combined with RT. However, 12% (14/118) of patients who did not undergo systemic chemotherapy in Mitsudo’s study developed distant metastasis [20,21]. These results suggest that high-dose systemic chemotherapy might decrease the risk of distant metastases in locally advanced tongue SCC.

Figure 4 shows that the cumulative cisplatin dose with IACT required for tumor control did not correlate with the tumor size. Of the five patients with local recurrence, four had tumor relapse within the drug perfusion area of IACT; three had relatively small tumors of approximately 10 cm^3^, which did not respond with cisplatin 500–620 mg for 7–10 cycles; and one was administered additional docetaxel 75 mg in four fractions. In this study, three patients died of local failure. They received radiation doses of 70 Gy, 61 Gy, and 57.6 Gy to the primary site and cisplatin doses of 622.5 mg, 620 mg, and 540 mg, respectively. The diseases in these patients were considered difficult to control even with higher doses of irradiation and cisplatin since systemic chemosensitivity was unfavorable. In contrast, in two patients who were excluded from the study, tongue tumors almost disappeared after treatment with only CRT without IACT. These results show that the chemosensitivity of patients with advanced tongue cancer greatly varies.

ECAS enabled us to remarkably improve IACT to select multiple feeding arteries. As a result, the treatment results were greatly improved [6]. However, the necessity for novel strategies for patients resistant to IACT must be explored.

This study has some limitations. First, it was a retrospective, single-center study with a small number of participants; therefore, it had inherent biases. However, the authors attempted to overcome this issue by including a homogenous study population with only stage III–IV tongue SCC patients. Thus, the statistical tests would not be able to identify significant relationships within the dataset. A larger sample size could have generated more accurate results. Second, the follow-up period was short. Therefore, the follow-up of the study cohort is still ongoing to further examine long-term prognosis and late toxicities.

## 5. Conclusions

This is the first clinical study to demonstrate that the ECAS for retrograde IACT is safe and highly effective for stage III–IV tongue cancer. Survival rates were not inferior to those of surgery, and no serious adverse effects associated with the procedure, i.e., catheter-related infections and cerebrovascular accidents, were observed. We believe that IACT using ECAS can be a promising organ-preserving treatment option for locally advanced tongue cancer.

## Figures and Tables

**Table 1:** Patient characteristics.

Characteristics	No. of Patients (%)
Gender	
Male	23 (74)
Female	8 (26)
Age (years)	
Range	25–76
Median	49
T classification	
T3	13 (42)
T4a	17 (55)
T4b	1 (3)
N classification	
N0	5 (16)
N1	4 (13)
N2b	14 (45)
N2c	7 (23)
N3b	1 (3)
Stage classification	
III	5 (16)
IVA	24 (77)
IVB	2 (7)
Reasons for not performing surgery	
Refusal	29 (94)
Inoperable disease	2 (6)
Total	31 (100)

**Table 2:** Adverse events (NCI-CTCAE v.4.0).

Toxicities		No of Patients by Toxicity Grade (%)		
	Grade 1	Grade 2	Grade 3	Grade 4
Acute				
Neutropenia	4 (13)	8 (26)	10 (32)	2 (7)
Anemia	9 (29)	17 (55)	3 (10)	0
Thrombocytopenia	21 (68)	2 (7)	3 (10)	0
Nausea	3 (12)	9 (29)	4 (13)	_
Oral mucositis	2 (7)	14 (45)	14 (45)	0
Dry mouth	11 (36)	12 (39)	3 (10)	_
Dysphagia	3 (10)	14 (45)	0	0
Radiation dermatitis	27 (87)	4 (13)	0	0
Renal failure	0	0	0	0
Fever	10 (32)	1 (3)	1 (3)	0
Catheter related infection	0	0	0	0
				
Late				
Ostenoradionecrosis	0	0	0	0

National Cancer Institute Common Terminology Criteria for Adverse Event v4.0.

**Table 3:** Univariate analysis of patient’s characteristics and treatment factors.

Variables	Level	No.	Overall Survival HR (95%CI)	*p* Value	Progression-Free Survival HR (95%CI)	*p* Value	Local Control HR (95% CI)	*p* Value
Age (y)	<50 ≥50	15 16	1 3.71 (0.41–33.25)	0.241	1 7.74(0.95–63.19)	0.056	undefined	0.999
Sex	Male Female	23 8	1 2.49 (0.41–15.02)	0.322	1 1.96(0.47–8.20)	0.359	1 1.86 (0.31–11.16)	0.496
T classification	3 4	13 18	1 1.09 (0.18–6.52)	0.929	1 0.67(0.17–2.69)	0.573	1 0.40 (0.07–2.41)	0.317
N classification	0 or 1 ≥2	9 22	1 0.40 (0.07–2.42)	0.318	1 0.30(0.08–1.22)	0.093	1 1.38 (0.15–12.35)	0.774
Stage	III IVA-IVB	5 26	1 0.48 (0.05–4.36)	0.514	1 0.39 (0.08–1.97)	0.253	1 0.50 (0.06–4.57)	0.543
Tumor volume (cm^3^)	<20 ≥20	15 16	1 0.60 (0.10–3.59)	0.575	1 0.50 (0.12–2.10)	0.344	1 0.56 (0.09–3.35)	0.524
RT dose (Gy)	<56 ≥56	13 18	1 3.32 (0.37–29.72)	0.284	1 1.22 (0.29–5.10)	0.789	1 3.01 (0.34–26.94)	0.325
Systemic chemotherapy (times)	1 2	6 25	1 0.25 (0.04–1.52)	0.133	1 0.19 (0.05–0.76)	0.019	1 0.32 (0.05–1.94)	0.216
Regimen of systemic chemotherapy	FP TPF	16 15	1 0.57 (0.10–3.44)	0.543	1 0.29 (0.06–1.44)	0.129	1 0.59 (0.10–3.54)	0.563
CDDP dose by IACT (mg)	<550 ≥550	15 16	1 0.52 (0.09–3.14)	0.478	1 0.86 (0.21–3.43)	0.826	1 0.58 (0.10–3.45)	0.546
IACT (times)	<7 ≥7	10 21	1 0.60 (0.10–3.62)	0.580	1 1.41 (0.28–7.00)	0.673	1 1.83 (0.20–16.38)	0.590

**Table 4:** Multivariate analysis of patient’s characteristics and treatment factors.

Progression-Free Survival Factor	Level	HR (95%CI)	*p* Value
Age (y)	<50 ≥50	1 5.25 (0.63–43.78)	0.126
N classification	0 or 1 ≥2	1 0.46 (0.11–1.86)	0.278
Systemic chemotherapy (times)	1 2	1 0.31 (0.08–1.25)	0.099

**Table 5:** Summary of treatment outcomes of patients with locally advanced tongue SCC.

Study (Publish)	Year of Collection	Sample Size	Stage	Treatment	Survival
Fan [22] (2007)	1995–2002	201	III, IV	OP + CRT	48% (3-y OS) (III: 64%, IV: 37%)
Sakamoto [23] (2011)	1996–2007	32	III, IV	OP ± CRT	III:77.1% (5-y DFS), IV:39.7% (5-y DFS)
Suzuki [24] (2014)	2000–2010	89	III, IV	OP ± RT	III:71.5% (5-y OS), IV:61.5% (5-y OS) III:78.6% (5-y CSS), IV:69.1% (5-y CSS)
Mroueh [5] (2017)	2005–2009	90	III, IV	OP ± RT/CRT	61% (5-y OS) III:69% (5-y DSS), IV:51% (5-y DSS)
Yasumatsu [25] (2020)	2007–2016	46	III, IV	OP ± CRT	III:70% (3-y DSS), IVA:64.2% (3-y DSS)
Kravets [19] (2020)	2004–2013	114	III, IV	OP + RT/CRT	57% (5-y OS), 56.5% (5-y DFS)
Oikawa [26] (2021)	2008–2017	89	III, IV	OP ± CRT	III:84.1% (5-y DSS), IV:79.0% (5-y DSS)
Ansarin [27] (2021)	2000–2018	353	III, IV	OP ±CRT	55% (5-y OS), 60% (5-y CSS), 50% (5-y DFS)
Fuwa [6] (2008)	1993–2002	88	III, IV	IACT + CRT	57 % (3-y OS), 72 % (3-y LC)
Doweck [28] (2008)	1993–2000	22 oral cavity *	III, IV	IACT + RT	37% (5-y OS), 69% (5-y LC)
Takayama [21] (2016)	2009–2012	33	III, IV	IACT + CRT by protonbeam	87.0% (3-y OS), 74.1% (3-y PFS), 86.6% (3-y LC)
Mitsudo [20] (2018)	2006–2015	95	III, IV	IACT + RT	III:94.7% (3-y OS), IV:64.9% (3-y OS) III:89.7% (3-y LRC), IV:72.1% (3-y LRC)
Nomura (present study)	2015–2021	31	III, IV	IACT + CRT	81.6% (3-y OS), 74.2% (3-y PFS), 83.4% (3-y LC)

* Subsite unknown. Abbreviations: IACT, intra-arterial chemotherapy; OP, operation; RT, radiotherapy; CRT, chemoradiotherapy; OS, overall survival; DSS, disease specific survival; CSS, cause specific survival; DFS, disease-free survival; LC, local control rate; LRC, locoregional control rate.

## Data Availability

The data presented in this study are available on request from the corresponding author.
